# The gut-brain axis: potential reshaping the future of anti-NMDAR encephalitis treatment

**DOI:** 10.3389/fimmu.2025.1668222

**Published:** 2025-11-28

**Authors:** Changchang Shen, Yinyin Xie, Yi Xie, Lina Wang, Aoya Han, Xinru Zhou, Shijie Zhang, Xili Fu, Nanchang Xie

**Affiliations:** Department of Neurology, The First Affiliated Hospital of Zhengzhou University, Zhengzhou, China

**Keywords:** anti-NMDAR encephalitis, gut microbial compositional shift, metabolites, blood-brain barrier, therapeutic potential

## Abstract

Anti-N-methyl-D-aspartate receptor (NMDAR) encephalitis is a type of autoimmune encephalitis with a high disability rate. However, its pathogenesis remains unclear and warrants further elucidation to facilitate the development of effective treatment strategies. The gut microbiota can increase the permeability of the intestinal and blood-brain barriers by altering the levels of beneficial bacterial metabolites and pro-inflammatory factors. These alterations facilitate the migration of inflammatory factors and pathogenic autoantibodies to the central nervous system, thereby affecting the progression of anti-NMDAR encephalitis. Therefore, interventions targeting the gut microbiota may be effective for regulating the pathogenesis of anti-NMDAR encephalitis. In this review, we discuss the association between an altered gut microbiota and associated changes in metabolite levels in patients with anti-NMDAR encephalitis. To our knowledge, this review is the first to outline the effects of the gut microbiota on cytokine levels in the blood and cerebrospinal fluid of patients with anti-NMDAR encephalitis, which may provide a new therapeutic perspective for this fatal disease.

## 1 Introduction

Anti-N-methyl-D-aspartate receptor (NMDAR) encephalitis, the most common type of autoimmune encephalitis, is caused by antibodies targeting the glutamate ionotropic receptor NMDA type subunit 1 (GluN1) of the NMDAR (1, 2). This disease was first identified in 2007 by Josep Dalmau et al., and its diverse clinical manifestations include psychiatric disturbances, seizures, cognitive dysfunction, and memory impairment (3). These symptoms are typically alleviated with first-line therapies (such as glucocorticoids and plasmapheresis) and second-line therapies (such as rituximab and cyclophosphamide) (4). However, some refractory patients respond poorly to conventional immunotherapies and exhibit rapid disease progression, leading to higher rates of disability and mortality (5). This demonstrates the urgent need for elucidation of the pathogenesis of anti-NMDAR encephalitis to provide a theoretical basis for breakthroughs in clinical treatment, thereby achieving dual improvements in patient neurological function and survival quality.

The gut microbiota has been implicated in the pathogenesis of anti-NMDAR encephalitis in recent years (6–11). The gut microbiota is a community of microorganisms primarily composed of bacteria that reside in the human gastrointestinal tract. It helps maintain the stability of the intestinal environment by synthesizing vitamins, regulating the immune system, and preserving the intestinal mucosal barrier, among other functions. In addition, it is closely associated with human health and disease (12). The gut microbiota may be involved in the pathogenesis of anti-NMDAR encephalitis through the “gut-brain axis.” The “gut-brain axis” refers to the bidirectional communication system between the gut and brain and can affect brain function by transmitting information through various pathways (such as metabolites, vagus nerves, and cytokines) (13). Changes in the gut microbiota can transmit signals through the “gut-brain axis” to regulate the immune and nervous systems, thereby influencing the pathogenesis of anti-NMDAR encephalitis. Therefore, modulation of the gut microbiota may be an effective treatment for this disease (9).

This review outlines the alterations in the gut microbiota of patients with anti-NMDAR encephalitis and identifies the potential association between these changes and anti-NMDAR encephalitis. In this review, we further discuss the application of the gut microbiota in the prediction of the course and treatment of anti-NMDAR encephalitis, which may contributes to considerable advancements in personalized treatment and clinical prognostic evaluation of this disease.

## 2 Characterization of gut microbial compositional shifts in patients with anti-NMDAR encephalitis

### 2.1 Alterations in the gut microbiota of patients with anti-NMDAR encephalitis

The gut microbiota of patients with anti-NMDAR encephalitis differs from that of healthy individuals. Specifically, newly diagnosed patients with anti-NMDAR encephalitis exhibit reduced fecal microbial diversity compared with healthy controls(**Table 1**) (7, 8, 11). This reduction in microbial diversity is consistent with observations in various other diseases such as non-alcoholic fatty liver disease, type 2 diabetes mellitus, hypertension and Alzheimer’s disease (14–17). However, Gong et al. reported contrasting findings, detecting higher gut microbiota diversity in patients with anti-NMDAR encephalitis than in healthy controls(**Table 1**) (6). Greater bacterial diversity is associated with healthier host status, as diverse microbial communities can deplete the nutrients required for pathogens to colonize the gut, thereby protecting the host from exogenous microbial invasion (18). Herken et al. presented a third outcome, indicating no significant difference in gut microbiota diversity between patients with anti-NMDAR encephalitis and healthy controls (10).

**Table 1:** Alterations in the gut microbiota of patients with anti-NMDAR encephalitis.

Study(Year)	Human participants (n)	Alteration of the gut microbiota	Reference
Gong, et al. (2019)	AE(30) vs. HC(12)	higher species richness **Genus^1^:** *Fusobacterium*, *Streptococcus*, *Escherichia‐Shigella*, *Bacteroides*↑; *Prevotella_6*, *Bifidobacterium*, *Faecalibacterium*↓	(6)
Herken, et al. (2019)	AE(23)vs. HC(24)	**Genus:** *Clostridium XVIII*, *Clostridium IV*, *Oscillibacter*, *Prevotella*, *Blautia*↑(acute phase, not significant after correction for multiple testing)	(10)
Chen,et al.(2020)	AE(40)vs. HC(54)	lower α-diversity **Phyla^2^:** *Proteobacteria*↑; *Firmicutes*↓ **Genus:** *Bacteroides*, *Enterococcus*, *Escherichia*, *Veillonella*, *Streptococcus*, *Dorea*, *Scardovia*, *Clostridium*↑; *Faecalibacterium*, *Roseburia*, *Lachnospira*, *Ruminococcus*, *Dialister*, *Coprococcus*, *Collinsella*, *Anaerostipes*↓	(7)
Gong,et al. (2022)	AE(58)vs. HC(49)	lower overall diversity **Genus:** *Enterococcus*,*Fusicatenibacter*,*Sellimonas*↑; *Bacteroides*, *Anaerostipes*, *Megamonas*, *Ruminococcus*, *Butyricicoccus*, *Faecalibacterium*↓	(8)
Wei, et al. (2022)	AE(10)vs. HC(10)	lower α-diversity **Family^3^:** *Streptophyta*,*Lactobacillaceae*,*Gemellaceae*,*Gemellales*↑; *Halomonas*,*Halomonadaceae*,*Butyricimonas*,*Odoribacteraceae*↓ **Genus:** *Prevotella*,*Lactobacillus*,*Actinomyces*↑; *Oceanospirillales*,*Faecalibacterium*↓	(11)

↑, higher abundance; ↓, lower abundance. ^1^A refined classification level comprising species with high genetic and functional similarity. ^2^A broad taxonomic rank that groups bacteria based on major structural and genetic characteristics. ^3^A mid-level taxonomic rank that includes related genera sharing evolutionary traits.

Differences among research findings may be attributed to the sequencing technologies employed: some studies utilize 16S rRNA gene sequencing, which is more suitable for capturing microbial diversity at the genus or partial species level; whereas others adopt metagenomic sequencing, offering higher resolution at the species or even strain level (19). Therefore, different sequencing approaches may lead to divergent interpretations of gut microbiota diversity and its biological significance. In addition, small sample sizes (e.g., some studies included fewer than 30 cases) may reduce statistical power and hamper the ability to effectively detect differences between patients and healthy controls. Additionally, the gut microbiota is highly complex. Thus, a single diversity index can only quantify the species composition and abundance of microorganisms, potentially failing to reflect the functional state of the microbiome. In certain cases, variations in the abundance of specific bacterial genera may have greater pathological significance than overall microbial diversity (20). This viewpoint was corroborated by Gong et al. and Wei et al., who reported that the composition of the fecal microbiota of patients with anti-NMDAR encephalitis significantly differed from that of healthy controls (6, 8, 11). Furthermore, the abundance of some commensal bacterial genera, such as *Faecalibacterium*, was significantly decreased in the patients (**Table 1**). As a dominant component of the gut microbiota, *Faecalibacterium* produces butyrate (21), which can not only suppress intestinal inflammatory responses by regulating immune cell differentiation (22), but also maintain the integrity of the intestinal barrier by enhancing intercellular tight junctions of intestinal epithelial cells (23). A decrease in the abundance of such beneficial genera can disrupt a healthy gut state and increase susceptibility to diseases. Similarly, Chen et al. observed significant differences in the composition of fecal microbiota between patients with anti-NMDAR encephalitis and healthy controls (**Table 1**) (7). These findings suggest that changes in the gut microbiota are a concomitant phenomenon of disease occurrence and may play an important role in disease pathogenesis.

### 2.2 Varying characteristics of the gut microbiota at different disease stages

The composition of the gut microbiota differs across the various stages of anti-NMDAR encephalitis (6). An increased abundance of *Fusobacterium* was observed in the gut microbiota of patients in the acute phase (**Table 1**). Toxins secreted by *Fusobacterium* may exacerbate neurological damage by inducing macrophage apoptosis and stimulating the release of proinflammatory cytokines (24). In contrast, patients in the relapse phase exhibited an increase in the abundance of *Streptococcus*, which can interfere with the host’s immune recognition mechanism by producing specific endopeptidases. This consequently reduces the immune response, which enables long-term survival within the host and triggers recurrent inflammatory episodes (25). This pattern of microbiota evolution provides important insights into the mechanisms underlying disease progression.

The composition of the gut microbiota is closely associated with the clinical features of anti-NMDAR encephalitis. The abundance of *Alistipes* spp. is higher in the gut microbiota of patients with psychiatric symptoms than in those without psychiatric symptoms (**Table 1**) (7). As indole-positive bacteria, *Alistipes* spp. promote the conversion of tryptophan to kynurenine by activating the indoleamine 2,3-dioxygenase, which reduces the amount of tryptophan that can be converted to 5-hydroxytryptamine (5-HT) (26). 5-HT is an important monoamine neurotransmitter in the central nervous system, and a decrease in its levels in the synaptic gap attenuates the activation of postsynaptic membrane 5-HT receptors (27), which destabilizes the neural loop of mood regulation and leads to psychiatric symptoms such as anxiety (26). These studies demonstrate differences in the microbiota of patients with anti-NMDAR encephalitis characterized by different clinical features, thereby offering new perspectives on the role of gut microbiota in the pathogenesis of disease. However, the small sample size precludes further elucidation of the relationship between microbiota abundance differences across groups and 5-HT levels. Furthermore, the lack of animal studies limits the validation of underlying mechanisms. Therefore, increased sample sizes and large-scale animal experiments are required to further explore their clinical significance.

## 3 Mechanisms by which the gut microbiota affects anti-NMDAR encephalitis

### 3.1 Immune homeostasis dysregulation

Gut microbial compositional shifts can enhance autoimmune responses against the central nervous system by altering immune cell differentiation and promoting the release of pro-inflammatory factors. For example, transplantation of the gut microbiota of patients with anti-NMDAR encephalitis into germ-free mice significantly increased the proportion of Th17 cells in the spleen and lamina propria of the small intestine (7). This was accompanied by an increase in the levels of pro-inflammatory cytokines such as IL-17 and IL-21 (9). These findings suggest that the gut microbiota of patients may be enriched in bacterial genera that can strongly induce Th17 cell differentiation, such as segmented filamentous bacteria (SFB). After colonizing the intestinal epithelial cells, SFB trigger the secretion of serum amyloid A by epithelial cells, thereby stimulating dendritic cells in the intestinal lamina propria to secrete IL-23 (28), thereby driving the differentiation of naïve CD4^+^ T cells into Th17 cells (29).

In addition to acting on intestinal epithelial cells, the gut microbiota can indirectly induce Th17 cell proliferation through metabolites. Both Gong et al. and Chen et al. reported an increased abundance of *Bacteroides* in patients with anti-NMDAR encephalitis, which may lead to elevated levels of succinate (30). This metabolite can promote the migration of dendritic cells to lymph nodes by binding to the Sucnr1 receptor, where the dendritic cells can activate and cause the expansion of Th17 cells (31). Moreover, as one of the major short-chain fatty acids (SCFAs) produced by the gut microbiota, butyrate enhances the acetylation of Forkhead box P3 (Foxp3+) proteins by inhibiting the deacetylation activity of histone deacetylase (HDAC) (32, 33), thereby promoting the differentiation and function of Treg cells (34). The reduced abundance of butyrate-producing bacteria such as *Faecalibacterium prausnitzii* in patients with anti-NMDAR encephalitis leads to decreased butyrate levels. This reduces the number of Treg cells (35), which limits the inhibitory effect on Th17 cells, thereby promoting their differentiation and increasing the Th17 cell population.

The abnormal proliferation of Th17 cells further results in the excessive secretion of pro-inflammatory cytokines such as IL-17 and IL-21. IL-17 binds to its highly expressed receptors on hippocampal Cornu Ammonis 1 (CA1) neurons, subsequently activating intracellular p38 mitogen-activated protein kinase (p38 MAPK). This disrupts hippocampal long-term potentiation (LTP), resulting in memory impairment (36). Contrastingly, IL-21 binds to its receptors on the surface of B cells, thereby activating downstream Janus kinase 1 (JAK1) kinase and phosphorylating STAT3 (37). The phosphorylated STAT3 binds to the promoter region of B-cell lymphoma 6 (Bcl-6) and increases the expression of Bcl-6, which then inhibits the expression of pro-apoptotic genes and promotes the formation of germinal center B cells (38). This enables the survival and differentiation of autoreactive B cells into plasma cells that secrete antibodies against the patient’s NMDAR (39).

Gong et al. reported a significant increase in the levels of cytokines, such as IL-6, IL-1, and TNF-α, in patients with anti-NMDAR encephalitis compared to healthy controls. Moreover, the levels of these cytokines were significantly elevated in patients with severe anti-NMDAR encephalitis and slightly increased in those with moderate disease presentation, whereas these cytokines were suppressed in healthy controls (8). The association between cytokine levels and disease severity further confirmed the effect of immune dysregulation on anti-NMDAR encephalitis.

### 3.2 Reduction in neurotransmitter synthesis

Neurotransmitters serve as carriers of synaptic communication and regulate cognitive, emotional, and motor functions by mediating the signal transmission between neurons (40). The synthesis, release, and metabolism of neurotransmitters or their precursors are not only regulated by neurons but are also closely related to metabolites of the gut microbiota (41). This finding opens new avenues for understanding neurological diseases such as anti-NMDAR encephalitis. Gut microbial compositional shifts with anti-NMDAR encephalitis may regulate the metabolism of neurotransmitter precursors through metabolites such as tryptophan, thereby contributing to the pathology of the disease. Tryptophan can cross the blood-brain barrier (BBB) into the brain (42), where it is synthesized into 5-HT and kynurenine (43, 44), This results in competition between the two synthesis pathways.

In patients with anti-NMDAR encephalitis, the increased abundance of *Bacteroides* may disrupt the balance of tryptophan metabolism, redirecting part of the tryptophan from the 5-HT pathway toward kynurenine metabolism (45). This metabolic imbalance results in dual neural impairments. The increased kynurenine can be further metabolized into quinolinic acid, which acts as an antagonist of NMDAR. Furthermore, the increased level of quinolinic acid exerts an antagonistic effect on NMDAR, leading to cognitive dysfunction in patients (46). 5-HT, a key neurotransmitter in the regulation of emotion, can alleviate anxiety by activating 5-HT1A receptors to suppress amygdala excitation (47, 48). It also plays an important role in learning and memory by activating 5-HT4 receptors to promote LTP in the hippocampus (49). Therefore, a reduction in 5-HT levels caused by tryptophan metabolic imbalance may induce anxiety and impair the learning and memory of patients.

The gut microbiota regulates the metabolism of neurotransmitter precursors in the host and produces various metabolites with neurotransmitter activity. For example, *Bifidobacterium* spp. can convert glutamate in the gut into γ-aminobutyric acid (GABA) (50). Although GABA has low permeability across the intestinal and blood-brain barriers, it may exert local effects on the vagus nerve, thereby indirectly affecting the central nervous system (41). As inhibitory neurotransmitters, GABA receptors facilitate the rapid influx of chloride ions by binding to chloride channels, leading to hyperpolarization of the neuronal membrane potential and inhibition of neuronal excitability (51).

In patients with anti-NMDAR encephalitis, a reduction in the abundance of *Bifidobacterium* spp. within the gut leads to decreased GABA synthesis, thereby weakening its inhibitory effect on neuronal excitability. This causes excessive neuronal excitation, which might result in epilepsy attacks (52). Moreover, a reduction in GABA levels can enhance acetylcholine release through the cholinergic system (53), leading to the activation of nicotinic acetylcholine receptors. The ligand-gated cation channels subsequently increase the influx of Na^+^ and Ca²^+^ (54). This leads to depolarization of the postsynaptic cell, which enhances neuronal excitability (55). Sustained neuronal discharges resulting from this excessive increase in excitability may also contribute to the onset of epileptic seizures.

### 3.3 Reduced BDNF expression

Brain-derived neurotrophic factor (BDNF) is an important neurotrophic substance in the central nervous system, which binds to Tropomyosin receptor kinase B (TrkB) receptors on postsynaptic neurons to promote synaptic strengthening and the formation of new synapses (56), consequently ensuring neuronal survival (57). It is closely linked to the pathological mechanisms of various neurological disorders such as schizophrenia (58). Moreover, BDNF can induce the dissociation of heterogeneous nuclear ribonucleoprotein K (hnRNPK) from the mRNA of Proline-rich tyrosine kinase 2 (Pyk2), thereby initiating the translation of Pyk2. This process ensures the local synthesis of Pyk2 at the synapse, leading to an increased abundance of NMDAR at the synaptic site (59).

The expression and function of BDNF in patients with anti-NMDAR encephalitis may be affected by the gut microbiota and its metabolites. Metabolites such as SCFAs and secondary bile acids produced by the gut microbiota can reach the central nervous system throughs systemic circulation, thereby regulating the synthesis and release of BDNF (60). As a typical SCFA, sodium butyrate is an important regulator of histone acetylation. By inhibiting histone deacetylases, it promotes histone H3 acetylation at the promoter region of the BDNF gene. Acetylation relaxes the chromatin structure and facilitates transcription factor access to the promoter region, thereby improving the transcription efficiency of BDNF and increasing its synthesis (61). Furthermore, butyrate can bind to G protein–coupled receptors, thereby activating the intracellular extracellular signal−regulated kinase (ERK) signaling pathway, which leads to the phosphorylation of cyclic adenosine monophosphate (cAMP) response element-binding protein (CREB) (62). The phosphorylated CREB then binds to the cAMP response element within the BDNF IXa promoter to initiate its transcription (63). However, a reduction in the abundance of *Faecalibacterium* in the gut of patients with anti-NMDAR encephalitis leads to decreased butyrate levels (6), thereby reducing BDNF transcription.

Secondary bile acids, generated from primary bile acids through the action of the gut microbiota (such as *Bacteroides* and *Enterococcus* species) (64), can activate Farnesoid X receptor (FXR) and promote the translocation of the CREB-regulated transcriptional coactivator 2 (CRTC2) from the nucleus to the cytoplasm (65). Under normal conditions, CRTC2 enters the nucleus and interacts with CREB to promote the transcription of *BDNF*. However, the activation of FXR disrupts this process, consequently preventing CRTC2 from binding to CREB in the nucleus. This reduces the CREB-mediated transcription of BDNF (66). Therefore, the increase in gut microbiota, such as *Bacteroides* and *Enterococcus* spp., in patients with anti-NMDAR encephalitis leads to elevated levels of secondary bile acids, ultimately potentially leading to the downregulation of BDNF expression (7). Decreased BDNF levels can lead to reduced neuronal survival, impaired synaptic plasticity, and a specific reduction in the abundance of NMDAR. This results in a range of symptoms, including cognitive and motor dysfunction (67).

### 3.4 Impairment of the gut and blood-brain barriers

**Figure 1:**
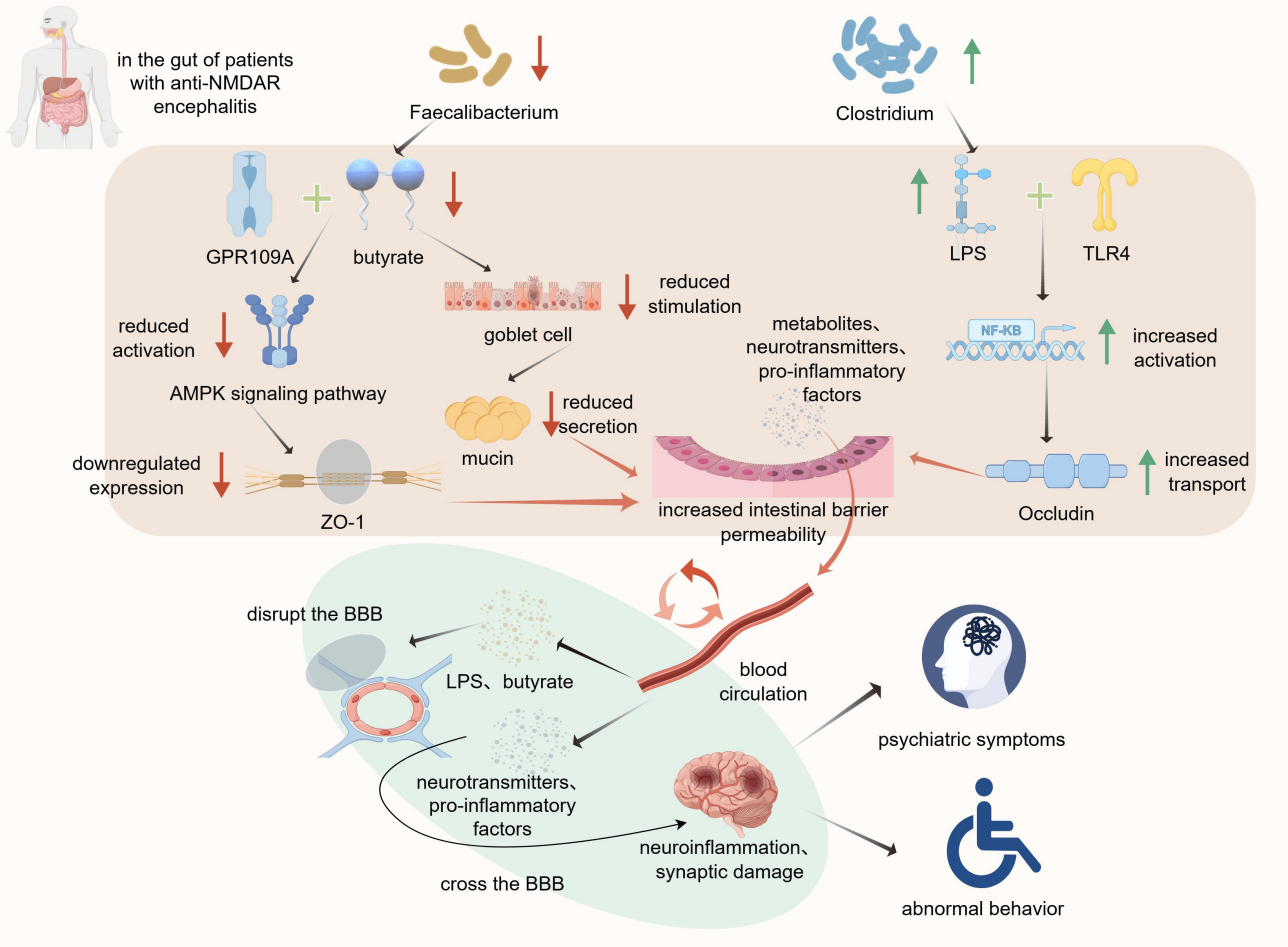
Altered gut microbiota composition contributes to intestinal barrier disruption and exacerbation of anti-NMDAR encephalitis symptoms. By Figdraw. Reduction in the abundance of *Faecalibacterium* decreases butyrate levels.

The gut barrier is composed of tightly arranged intestinal epithelial cells connected by tight junction proteins and covered by a mucus layer on the surface. This forms a robust physical barrier preventing the entry of bacterial toxins and inflammatory factors into the bloodstream (68). However, gut microbial compositional shifts, together with associated changes in metabolite levels, can compromise the integrity of the gut barrier (69). Gong et al. and Chen et al. identified gut barrier impairment in patients with anti-NMDAR encephalitis (7, 9), along with a reduction in the abundance of butyrate-producing bacteria such as *Faecalibacterium* in the gut (6). Butyrate deficiency exacerbates gut barrier damage through multiple pathways. First, butyrate maintains the proliferation of intestinal epithelial cells by generating ATP through β-oxidation (70). Additionally, butyrate can bind to the G-protein-coupled receptor 109A (GPR109A) in intestinal epithelial cells, activating the AMPK signaling pathway (71), which promotes the expression of tight junction proteins such as Zonula occludens−1 (ZO-1)(**Figure 1**) (72). Furthermore, butyrate stimulate goblet cells to secrete mucin (**Figure 1**), consequently maintaining the thickness of the mucus layer (73). Therefore, the decrease in butyrate levels caused by the reduced abundance of *Faecalibacterium* in patients leads to impaired proliferation of intestinal epithelial cells, disruption of tight junctions between cells, and thinning of the mucus layer. This increases the permeability of the intestinal barrier.

Lipopolysaccharides (LPS) play a critical role in intestinal mucosal dysfunction. The increased abundance of *Clostridium* in patients with anti-NMDAR encephalitis is accompanied by elevated levels of LPS (7). The LPS molecules penetrate the damaged mucus layer and bind to Toll−like receptor 4 (TLR4) receptors on the surface of intestinal epithelial cells, consequently activating the NF-κB pathway. This process promotes the translocation of tight junction proteins, such as Occludin and ZO-1 (**Figure 1**), thereby altering their distribution within the cell membrane and ultimately disrupting the intestinal barrier (74).

This reduces their binding to GPR109A, which inhibits the activation of the AMPK signaling pathway and lowers ZO-1 expression. The decline in butyrate levels also attenuates goblet cell stimulation, leading to decreased mucin secretion. Furthermore, the increased abundance of *Clostridium* elevates LPS levels, consequently enhancing its binding to TLR4. TLR4 activates the NF-κB signaling pathway and promotes Occludin translocation to increase intestinal barrier permeability. Subsequently, metabolites, neurotransmitters, and pro-inflammatory factors cross the intestinal barrier into the bloodstream, reach the BBB, compromise its integrity, and trigger neuroinflammation and synaptic damage, ultimately leading to a spectrum of psychiatric and behavioral symptoms associated with anti-NMDAR encephalitis.

Disruption of the intestinal barrier allows metabolites and pro-inflammatory factors to enter the bloodstream and reach the BBB (**Figure 1**). The BBB is primarily composed of brain capillary endothelial cells, basement membrane, and astrocytic end-feet (75). This highly selective barrier prevents the entry of harmful substances into the brain tissue and maintains a stable intracerebral environment. As an HDAC inhibitor, butyrate suppresses the activation of the NF-κB pathway, leading to downregulation of the expression of Matrix metallopeptidase 9 (MMP-9), an enzyme responsible for tight junction degradation. This ultimately prevents the degradation of tight junction proteins, such as ZO-1 and claudin-5 (76, 77), thereby protecting the BBB. Therefore, reduced butyrate levels increase the degradation of tight junction proteins. Additionally, upon reaching the BBB, LPS can increase its permeability through mechanisms similar to those observed in the intestinal barrier (78). Consequently, certain pro-inflammatory cytokines and neurotransmitters can penetrate the compromised BBB and affect the central nervous system, thereby exacerbating symptoms via persistent neuroinflammation and neurotransmitter dysregulation (**Figure 1**). This plays a crucial role in the pathogenesis of anti-NMDAR encephalitis. Gong et al. further suggest that the dual impairment of the intestinal and blood-brain barriers establishes a critical “gut-brain axis” pathway that jointly promotes disease progression (9).

## 4 Predictive and therapeutic value of the gut microbiota in anti-NMDAR encephalitis

### 4.1 Predicting clinical outcomes

The clinical manifestations of anti-NMDAR encephalitis are diverse, and patient prognoses vary significantly. Predicting clinical outcomes is crucial for the early identification of high-risk patients, as it allows the timely initiation of second-line immunotherapy. However, traditional clinical indicators such as the NEOS score have limited predictive capability (79). With advancements in multiomics technologies, integrated analyses combining metagenomics, metabolomics, and clinical phenomics of gut microbiota characteristics, microbial metabolites, and clinical features of patients offer a novel perspective for outcome prediction. Gong et al. revealed that patients with higher gut microbiota diversity did not experience relapses during follow-up. This suggests that patients with lower bacterial diversity have a higher risk of relapse than those with higher microbial diversity (8). Higher bacterial diversity is associated with smaller fluctuations in gut metabolite levels, allowing faster recovery and stabilization of gut function. This consequently reduces the likelihood of disease recurrence. Furthermore, analysis of the fecal bacterial composition by Gong et al. identified *Granulicatella* as a strong predictor of favorable clinical outcomes, possibly because its fermentation of carbohydrates produces lactic acid, which may lead to the phosphorylation-induced inactivation of Yes−associated protein (YAP) (80). This reduces the interaction between YAP and the p65 subunit of NF-κB, thereby inhibiting the NF-κB pathway, decreasing the release of pro-inflammatory cytokines such as TNF-α, and mitigating inflammation (81). In support of this hypothesis, patients with poor prognoses exhibit significantly lower *Granulicatella* abundance than those with better clinical outcomes.

Metabolomic analysis of patient feces and serum has identified key metabolites that can aid in the prediction of clinical outcomes in anti-NMDAR encephalitis. Fecal metabolites such as l-carnitine and lysophosphatidic acid (LPA), along with serum metabolites such as kynurenine and choline, have shown predictive potential. L-carnitine exerts neuroprotective effects in Alzheimer’s disease by restoring mitochondrial membrane potential and promoting normal mitochondrial dynamics (82). However, LPA can activate its receptor, LPA1, to induce pro-inflammatory effects and exacerbate neuroinflammation (83). In addition, it has been used to predict the development of autoimmune hepatitis and the response of patients with tumors to immunotherapy (84, 85). Both factors can affect clinical outcomes in patients with anti-NMDAR encephalitis. Additionally, serum kynurenine can be further metabolized into quinolinic acid, an NMDAR antagonist that affects cognitive function. Contrastingly, choline can cross the BBB and serve as a precursor of acetylcholine, playing a crucial role in the regulation of learning and memory. A more precise prediction of clinical outcomes can be achieved by integrating fecal and serum metabolite biomarkers with previously identified microbiota markers and the NEOS score. This refined approach offers reliable guidance for the early initiation of second-line immunotherapy (8). The superiority of this combined model further underscores the groundbreaking value of multi-omics integration, surpassing traditional single-indicator predictive methods.

### 4.2 Potential therapeutic value

Immunomodulatory therapy is the primary treatment for anti-NMDAR encephalitis (1, 4). Although this approach significantly improves patient survival, some individuals experience relapses after treatment. In addition, long-term use of immunosuppressants may lead to severe adverse effects such as infections (86). These therapies, which alleviate the symptoms by suppressing the immune system or eliminating autoantibodies, do not address the underlying mechanism of the disease. Recent advances in research on the “gut-brain axis” have provided new therapeutic possibilities for anti-NMDAR encephalitis. Germ-free mice receiving fecal microbiota transplantation (FMT) from patients with anti-NMDAR encephalitis exhibit behavioral deficits and cognitive impairments similar to those of donors, such as reduced exploratory behavior and diminished spatial memory and learning abilities (7). This suggests that the transplanted gut microbiota may induce central nervous system dysfunction by modulating immune homeostasis and neural signaling pathways in the new host, resulting in behavioral phenotypes resembling those of the donor. FMT, which involves the transfer of the gut microbiota from healthy donors to patients, such as beneficial bacterial strains that produce SCFAs, has the potential to restore the gut microbiome balance and serve as a novel therapeutic strategy (87). FMT has shown promise in autoimmune diseases such as inflammatory bowel disease (IBD), where it alleviates symptoms and reduces the frequency of acute flare-ups (88). Furthermore, it demonstrated significant efficacy in treating recurrent *Clostridioides difficile* infections (89). However, due to the highly individualized nature of gut microbiota composition, the effects of FMT may vary among individuals, leading to uncertainties in its therapeutic outcomes (90). Additionally, FMT carries the potential risk of viral transmission (91), consequently necessitating rigorous clinical and microbiological screening to minimize these risks.

As the regulation of disease mechanisms by the gut microbiota is largely mediated by metabolic products, recent studies have focused on more targeted metabolite-based interventions. This approach involves the direct supplementation of deficient microbial metabolites to alleviate disease symptoms (92). Supplementation with metabolites, such as SCFAs, has been successfully applied to improve colitis symptoms in mouse models (93). Moreover, recent studies have demonstrated that nicotinamide can alleviate neurological symptoms in mouse models of amyotrophic lateral sclerosis (94). Compared to FMT, metabolite-based intervention not only mitigates the biosafety risks associated with live bacterial transplantation, but also allows precise dosage control, making it a potentially safer and more controlled therapeutic strategy. Building on these advances, the treatment approaches for anti-NMDAR encephalitis are undergoing significant shifts. Therapeutic strategies are shifting from simple immunosuppression to gut-brain axis modulation and metabolic interventions, offering a more personalized treatment approach without increasing the risk of severe infections. This paradigm shift also provides a theoretical basis for the establishment of new approaches for the treatment of neuroimmune disorders.

## 5 Conclusion

Gut microbial compositional shifts play a significant role in the pathogenesis of anti-NMDAR encephalitis. Gut microbial compositional shifts are commonly characterized by reduced microbial diversity, a decline in the abundance of beneficial bacteria, overgrowth of pathogenic species, and disruptions in key metabolic pathways, including tryptophan metabolism and SCFAs synthesis. These alterations contribute to an immune homeostasis imbalance, abnormal neurotransmitter synthesis, and increased permeability of both the intestinal and blood-brain barriers, thereby exacerbating disease progression. These findings highlight the gut-brain axis as a **significant** factor in the pathogenesis of anti-NMDAR encephalitis and suggest new therapeutic approaches. FMT and supplementation with specific microbial metabolites offer promising strategies for restoring the gut microbiota balance and alleviating neuroinflammation. Future research should focus on larger-scale cohort studies, particularly those with prospective and longitudinal designs, to systematically and comprehensively record and control multiple potential confounding factors. These include dietary composition (such as nutrient intake, dietary fiber, and prebiotics), medication use (including immunosuppressants, antiepileptic drugs, and psychotropic agents), history of antibiotic or probiotic interventions, timing and duration of treatments during disease progression, as well as other lifestyle factors (such as sleep and physical activity). By tracking and stratifying these key variables, it will be possible to clarify how gut microbial compositional shifts specifically influence disease progression and to further validate microbiota-based therapeutic strategies. Elucidation of the mechanisms underlying anti-NMDAR encephalitis will not only provide insights into its pathological basis but also contribute to the identification of predictive biomarkers for disease progression. This will facilitate the development of more effective and personalized treatment strategies to overcome the limitations of conventional immunomodulatory therapies.
